# Integrative Analysis of CRISPR/Cas9 Target Sites in the Human *HBB* Gene

**DOI:** 10.1155/2015/514709

**Published:** 2015-03-31

**Authors:** Yumei Luo, Detu Zhu, Zhizhuo Zhang, Yaoyong Chen, Xiaofang Sun

**Affiliations:** ^1^Key Laboratory for Major Obstetric Diseases of Guangdong Province, Key Laboratory of Reproduction and Genetics of Guangdong Higher Education Institutes, The Third Affiliated Hospital of Guangzhou Medical University, Guangzhou 510150, China; ^2^Department of Biological Sciences, National University of Singapore, Singapore 117543; ^3^Department of Computer Science, National University of Singapore, Singapore 117543

## Abstract

Recently, the clustered regularly interspaced short palindromic repeats (CRISPR) system has emerged as a powerful customizable artificial nuclease to facilitate precise genetic correction for tissue regeneration and isogenic disease modeling. However, previous studies reported substantial off-target activities of CRISPR system in human cells, and the enormous putative off-target sites are labor-intensive to be validated experimentally, thus motivating bioinformatics methods for rational design of CRISPR system and prediction of its potential off-target effects. Here, we describe an integrative analytical process to identify specific CRISPR target sites in the human *β*-globin gene (*HBB*) and predict their off-target effects. Our method includes off-target analysis in both coding and noncoding regions, which was neglected by previous studies. It was found that the CRISPR target sites in the introns have fewer off-target sites in the coding regions than those in the exons. Remarkably, target sites containing certain transcriptional factor motif have enriched binding sites of relevant transcriptional factor in their off-target sets. We also found that the intron sites have fewer SNPs, which leads to less variation of CRISPR efficiency in different individuals during clinical applications. Our studies provide a standard analytical procedure to select specific CRISPR targets for genetic correction.

## 1. Introduction

Technologies to achieve precise gene correction in patient-specific induced pluripotent stem cells (iPSCs) are essential for stem cell-based tissue regeneration [1–4] and genetically matched disease modeling [5, 6]. Recently, the clustered regularly interspaced short palindromic repeats (CRISPR) system has emerged as a powerful tool for targeted genetic modification due to its easy programming, fast construction, robust efficiency, and multiplexable genomic editing, that is, editing of multiple target sites simultaneously [7, 8]. In this system, the CRISPR-associated 9 (Cas9) nuclease is directed by a synthetic single-guide RNA (sgRNA) consisting of a 20 nt guide sequence and an auxiliary transactivating sequence via base-pair complementarity to specific genome locus as a means to create DNA double-strand breaks (DSBs). The DSBs can be repaired either via error-prone nonhomologous end joining (NHEJ), which results in small insertions and deletions (Indels), or via homology-directed repair (HDR) if a double-strand donor DNA or a single-strand oligonucleotide template is present, which leads to precise sequence replacement [9]. Recent studies have exhibited successful correction of genetic defects in mouse zygotes [10] and in human adult stem cell organoids [11] using the CRISPR system. Compared with previous zinc finger nuclease (ZFN) and transcription activator-like effector nuclease (TALEN), the CRISPR system offers several advantages: simple, easy programming; fast, inexpensive construction; robustly efficient mutagenesis and multiplexed genome editing. Hence, the CRISPR system is suggested to be a powerful new tool for genetic correction via HDR in patient-specific iPSCs.

The DNA binding specificity of the CRISPR complex is dependent on the base-pair complementarity between the 20 nt sgRNA and the target genomic DNA sequence of interest that lies next to the 5′ end of a protospacer adjacent motif (PAM) matching the sequence NGG. The 1st nucleotide (numbered 1st to 20th in the 5′ to 3′ direction) of the sgRNA must remain G to avoid affecting expression driven by the U6 promoter. And the Cas9 nuclease will cleave at the 17th nucleotide of the recognition site [9]. Early studies have shown that Cas9-mediated cleavage can be abolished by single mismatches at the sgRNA:DNA target site interface [7, 8]; however, more systematic investigations revealed that the CRISPR system could cause substantial off-target effects in human cells which resulted from the binding between sgRNA and imperfectly complementary DNA sequences in the genome [12–14]. These off-target effects can be disastrous if they occur during the gene correction process in human cells. It is reported that the CRISPR system targeting a site near the sickle-cell mutation in the human* HBB* gene produces substantial off-target cleavages due to recognition of less perfectly complementary DNA sequences in the genome, especially at the neighboring homologous* HBD* gene, and even causes gross chromosomal deletions [15]. This problem will seriously hamper the extending application of CRISPR in genetic correction as many disease-associated genes have homologous family members. Although a double nicking strategy is newly developed to enhance the CRISPR cleavage specificity [16], it renders limited help in this situation as doubling the DNA recognition length does not necessarily confer more selectivity between two highly homologous genes. The great challenge remains in designing specific target sites that differentiate CRISPR cleavage activities between the two genes.

The enormous putative off-target sites are labor-intensive to be validated experimentally, thus motivating bioinformatics methods for rational design of sgRNA and prediction of its potential off-target effects. However, previous CRISPR off-target prediction tools focus mainly on protein-coding regions while neglecting the noncoding regions [14, 17–19]. In this study, we describe an integrative analytical process combining computational analyses of target uniqueness, off-target distribution in both exons and transcriptional factor binding sites (TFBS), and DNA variants information to identify specific CRISPR target sites in the* HBB* gene. To the best of our knowledge, this is the first CRISPR design method taking TFBS into consideration.

## 2. Materials and Methods

### 2.1. Phylogenetic Analysis

For phylogenetic analysis,* Homo sapiens* (human) and* Pan troglodytes* (chimpanzee) were chosen to represent mammals;* Gallus gallus* (chicken) and* Cairina moschata* (Muscovy duck) were chosen to represent Aves. The gene structures of the *α*- and *β*-globin gene clusters of* H. sapiens* and* G. gallus* are obtained from Ensembl (http://www.ensembl.org/) [20]. Their IDs are ENSG00000130656, ENSG00000188170, ENSG00000188536, ENSG00000196565, ENSGALG00000007458, ENSGALG00000007463, ENSGALG00000007468, ENSGALG00000017345, and ENSGALG00000017347. The protein function and sequence information of each gene are from the UniProtKB/Swiss-Prot DATABASE (http://www.ebi.ac.uk/uniprot) [21]. The protein sequences data are sorted by species and functions and then used for alignment and comparison. Their primary access numbers are P02008, P02042, P02100, P02144, P09105, P68871, P69891, P69892, P69905, P01935, P06347, P61772, P61920, P61921, P68873, P69907, Q6LDH1, P01994, P02001, P02007, P02112, P02127, P02128, P02197, P01987, P02003, P04243, P14260, and P14261. ClustalX 1.83 and Mega 3.0 were used to do complete alignment and construct the cladogram.

### 2.2. CRISPR Target Site and Off-Target Analysis

Firstly, to systemically analyze the CRISPR target sites in the region of interest, all candidate sites complying with the GN_19_NGG sequence pattern are searched using the web tool Cas9 Design (http://cas9.cbi.pku.edu.cn/) [17]. The candidate sites will be classified and numbered based on their locations in the exons and introns. Basic information such as sequence, genome position, and GC content of each candidate site will be included for further analysis.

Secondly, putative off-target sites of each candidate site with up to 3 target mismatches are searched against the human genome assembly hg19 using the web tool Optimized CRISPR Design (http://crispr.mit.edu/) [14]. To evaluate the potential off-target side effects, the distribution of these off-target sites in exons and transcription factor binding sites (TFBS) is searched as reported in UCSC genes and ENCODE TF ChIP-seq data of the UCSC Genome Browser (http://genome.ucsc.edu/) [22]. If binding sites of a certain TF were frequently found in the off-target set of a candidate site, the binding motif of this TF will be searched as reported in the JASPAR database (http://jaspardev.genereg.net/) [23].

Thirdly, as DNA variants in the target sequence might affect the CRISPR/Cas9 cleavage efficiency, the numbers of single nucleotide polymorphisms (SNPs) which overlapped with the candidate sites were searched as reported in dbSNP135 (http://www.ncbi.nlm.nih.gov/snp) [24].

### 2.3. CRISPR Plasmid Construction and T7E1 Assay

The pCas9/I2-1 CRISPR plasmid was generated by kinasing and annealing oligonucleotides containing the I2-1 guide strand plus sticky ends, ligating into the pX330 plasmid that contains a CHB promoter-driven Cas9 and a U6 promoter-driven chimeric single-guide RNA expression cassette (Addgene: 42230). The cleavage efficiency was measured using the T7 endonuclease I (T7E1) mutation detection assay. In brief, 10^6^ 293T cells/dish were plated onto 60 mm dishes and cultured in fibroblast medium 24 h prior to transfection. Cells were transfected with 2, 5, or 10 *μ*g of pCas9/I2-1 plasmid using Lipofectamine LTX (Invitrogen). 72 h after transfection, the genomic DNA samples were harvested. Short fragments flanking the endogenous I2-1 locus were amplified by PCR and subjected to T7E1 digestion at 37°C for 1 h. The digested fragments were then separated on agarose gels and quantified using ImageJ. The primer sets used can be found in Supplementary Information, Table S3, in Supplementary Material available online at http://dx.doi.org/10.1155/2015/514709.

## 3. Results

### 3.1. The Human *β*-Globin Gene Cluster Evolves by Tandem Duplication

**Figure 1:**
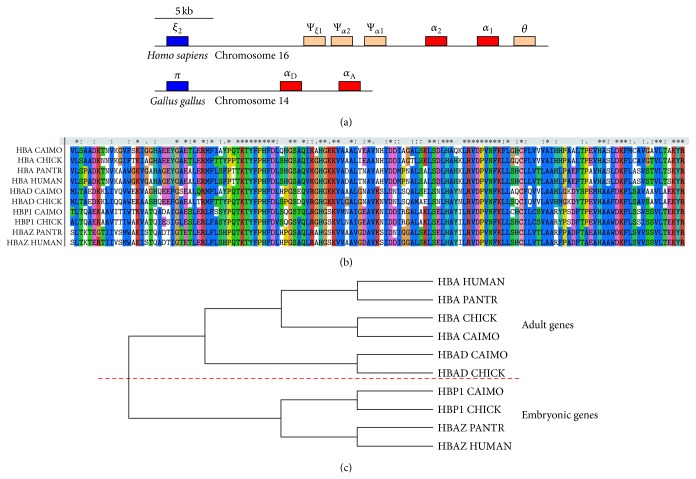
Phylogenetic analysis of *α*-like globins. (a) Structure model of the *α*-globin gene cluster in* Homo sapiens* and* Gallus gallus*. Adult genes are shown in red, embryonic genes in blue, and pseudogenes in pink. (b) Amino acid sequences and alignment result of *α*-like globins from* Homo sapiens* (human),* Pan troglodytes* (chimpanzee),* Gallus gallus* (chicken), and* Cairina moschata* (Muscovy duck). (c) Cladogram of *α*-like globin sequences. HUMAN,* H. sapiens*; PANTR,* P. troglodytes*; CHICK,* G. gallus*; CAIMO,* C. moschata*; HBA, hemoglobin subunit *α* (mammals) or *α*-A (Aves); HBAZ, hemoglobin subunit *ζ*; HBAD, hemoglobin subunit *α*-D; HBPI, hemoglobin subunit *π*.

Ahead of the CRISPR/Cas9 target site analysis, we investigate the evolution of *α*-like and *β*-like globins in Aves and mammals to understand why the human* HBB* gene has so many highly similar homologous genes. The alignment result and phylogenetic analysis of the protein sequences responding to the *α*-like globins are shown in Figure 1. The most significant finding is that the *α*-globins and the *α*-A globins, as well as the *ζ*-globins and the *π*-globins, are orthologous. Namely, the adult and embryonic *α*-like globins of Aves have the same ancestors as those of mammals, respectively. This result strongly supports the view that there had been globin gene tandem duplication happening before the genome duplication event [25]. The ancestral adult and embryonic *α*-like genes should have been existent and should have been expressed alternatively at that time.

**Figure 2:**
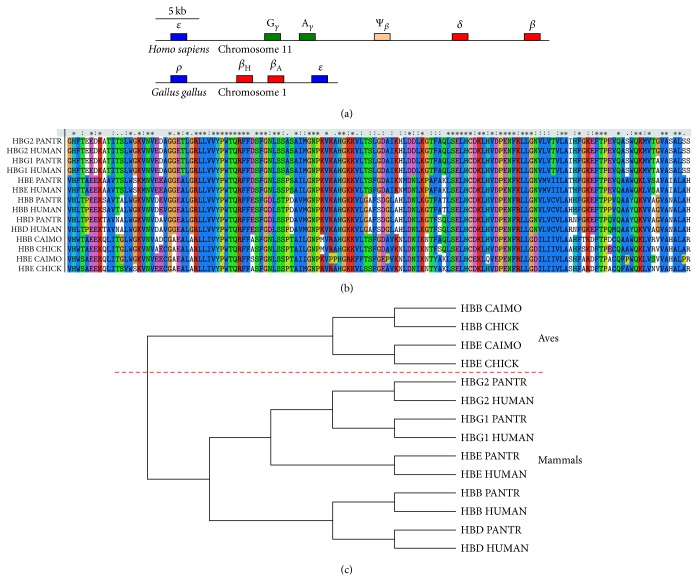
Phylogenetic analysis of *β*-like globins (a) Structure model of the *β*-globin gene cluster in* H. sapiens* and* G. gallus*. Adult genes are shown in red, fetal genes in green, embryonic genes in blue, and pseudogenes in pink. (b) Amino acid sequences and alignment result of *β*-like globins from* H. sapiens*,* P. troglodytes*,* G. gallus*, and* C. moschata*. (c) Cladogram of *β*-like globin sequences. HBB, hemoglobin subunit *β*; HBE, hemoglobin subunit *ε*; HBD, hemoglobin subunit *δ*; HBG1, hemoglobin subunit *γ*-1; HBG2, hemoglobin subunit *γ*-2.

The alignment result and phylogenetic analysis of the protein sequences responding to the *β*-like globins are shown in Figure 2. Obviously, the result fully supports the recent finding that the *β*-globin gene clusters of Aves and mammals are not orthologous [26]. It means that the *β*-globin gene clusters arose independently in mammals and Aves. This evidence also presents the evolutionary convergence of alternative expression. Moreover, it implies the importance of gene tandem duplication in new gene forming and the great effect of alternative expression in subtle regulation to an organism.

Collectively, these results are consistent with the* in trans* duplication theory [27] and can explain why there are multiple highly homologous genes in the human *β*-globin gene cluster as this region is quickly evolving by tandem duplication. This also motivates us to develop a new CRISPR target design method that can differentiate CRISPR/Cas9 cleavage activities between highly homologous genes, such as the human *β*-globin gene cluster.

### 3.2. Integrative Analysis for the CRISPR Target Sites in the Human* HBB* Gene

**Figure 3:**
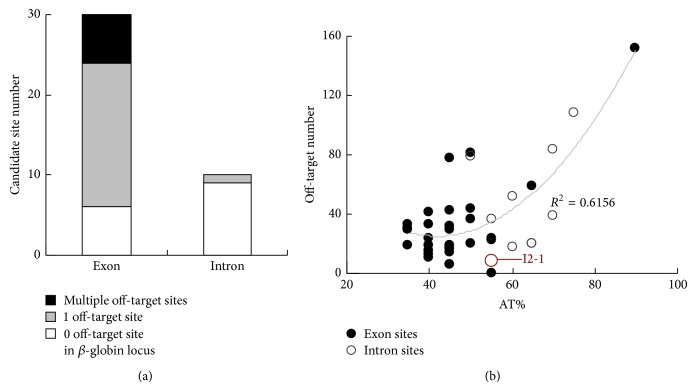
Uniqueness analysis of CRISPR target sites in* HBB* gene. (a) Statistics of candidate CRISPR target sites found in exons and introns of* HBB*. (b) Scatter plot showing the correlation between off-target numbers and AT% of each candidate site.

An integrative analytical process combining computational analyses of target uniqueness, off-target distribution, and DNA variants information was developed to identify specific CRISPR target sites for* HBB* gene correction. Firstly, we scanned the* HBB* gene region (hg 19, chr11: 5,246,696–5,248,301) for candidate CRISPR target sites based on the GN_19_NGG sequence pattern. A total of 40 candidate sites were found and numbered by locations in the 3 exons (E1/2/3-#) and 2 introns (I1/2-#) (Supplementary Information, Table  S1). Target uniqueness analysis showed that among the 30 sites found in exons, 18 sites had almost identical off-targets at HBD and 6 sites had one or more highly similar off-targets (with none or merely 1 nucleotide mismatch) at other homologous *β*-like globin genes (Figure 3(a)). In contrast, only 1 out of the 10 intron sites had an off-target site at* HBD* (Figure 3(a)). Thus, it seems that the intron region has less conserved homology sequences than the exon region.

Secondly, based on previous finding that the CRISPR system potentially tolerated 1–3 target mismatches [12], we investigated all the putative off-target sites in the human genome (hg19) of each candidate site with up to 3 mismatches (Supplementary Information, Table S2). Intriguingly, it showed that candidate sites with >75% AT content had much higher numbers of putative off-target sites (Figure 3(b)), which is probably due to lower sequence complexity. Adding to previous report stating that sgRNAs with too high or too low AT content were less effective against their targets [28], it is suggested to select target sites with moderate AT content.

**Figure 4:**
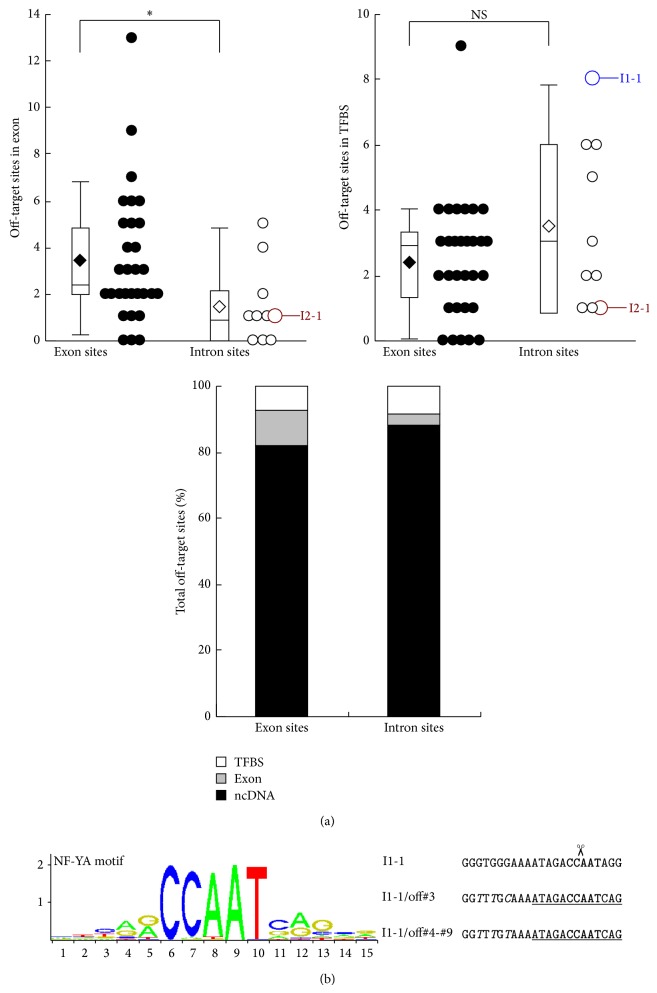
Off-target distribution analysis of CRISPR target sites in* HBB* gene. (a) Statistics of off-target sites located in exons, transcriptional factor binding sites (TFBS), or noncoding DNA (ncDNA). The box plots depict 10th, 25th, 50th, 75th, and 90th percentiles of the candidate sites. The diamond points in the boxes indicate the average off-target numbers. The scatter plots show relevant off-target numbers of each candidate site. (b) Identification of conserved NF-YA binding sites in off-target sites #3–#9 of the candidate site I1-1.

Furthermore, to evaluate the potential off-target effects, the numbers of off-target sites located in other exons and transcription factor binding sites (TFBS) validated by ChIP-seq data from the ENCODE Project were investigated. Averagely, both the exon and intron sites had approximately 8% off-target sites located in TFBS; however, the exon sites had significantly more off-target sites located in other exons, which is around 2.5 times of those of the intron sites (Figure 4(a)). Another interesting finding was that one of the intron sites, I1-1 (GGGTGGGAAAATAGACCAATAGG), had off-target sites abnormally enriched in TFBS of NF-YA, a CCAAT-binding protein. Further TFBS motif analysis showed that I1-1 contained a CCAAT sequence pattern, and 7 of its putative off-target sites, #3–#9, contained the binding motif of NF-YA (Figure 4(b)). These off-target sites were almost identical, with only 1 different nucleotide at #3, indicating that they belonged to highly conserved regulatory elements.

**Figure 5:**
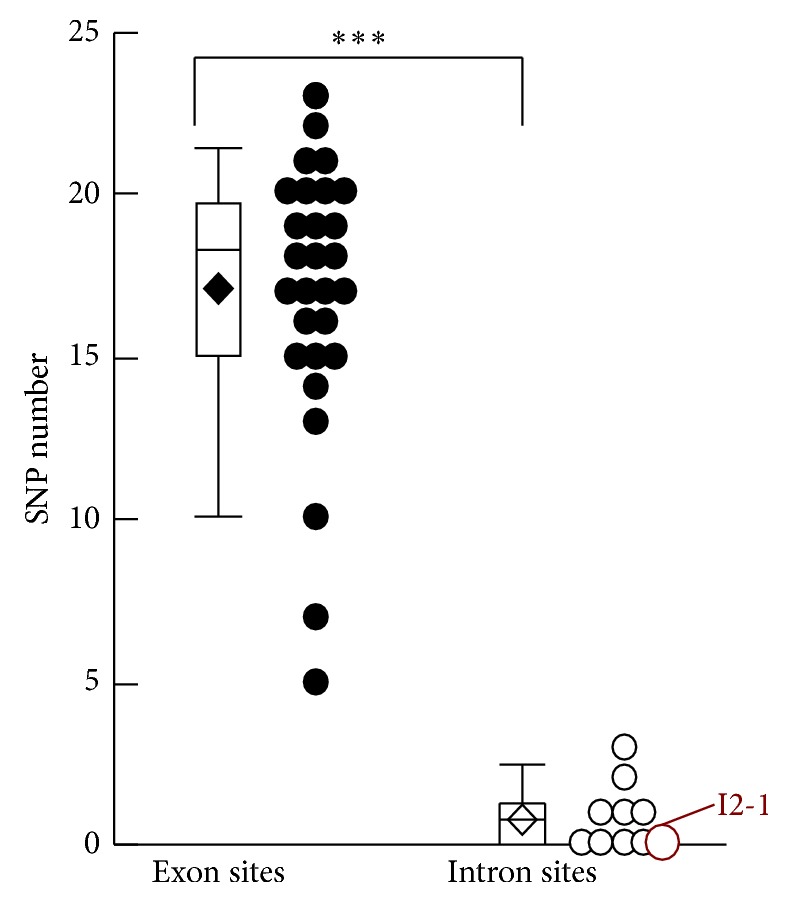
Single nucleotide polymorphism (SNP) analysis of CRISPR target sites in* HBB* gene.

Thirdly, the numbers of known single nucleotide polymorphisms (SNPs) in these candidate sites were also investigated by searching all overlapped SNPs as reported in dbSNP135. SNPs were not favored in this situation as they would result in variation of CRISPR cleavage activities between iPSC lines derived from different patients. The statistics showed that all exon sites contained more SNPs than intron sites, while there were 4 intron sites without any known SNP (Figure 5).

Finally, we found that target sites in introns had 2 advantages over those in exons: (i) less highly similar off-target sites at homologous genes; (ii) less off-target sites located in other exons. Moreover, to minimize possible off-target effects, the selection of target sites should also avoid (i) too high or too low AT content; (ii) containing common regulatory elements. Based on these criteria, we recommended I2-1 (GACGAATGATTGCATCAGTGTGG), an intron candidate site without any known SNP and with the fewest putative off-target sites across the genome, as the CRISPR target for* HBB* correction.

### 3.3. CRISPR System Efficiently Cleaves at the I2-1 Site without Significant Off-Target Effects

**Figure 6:**
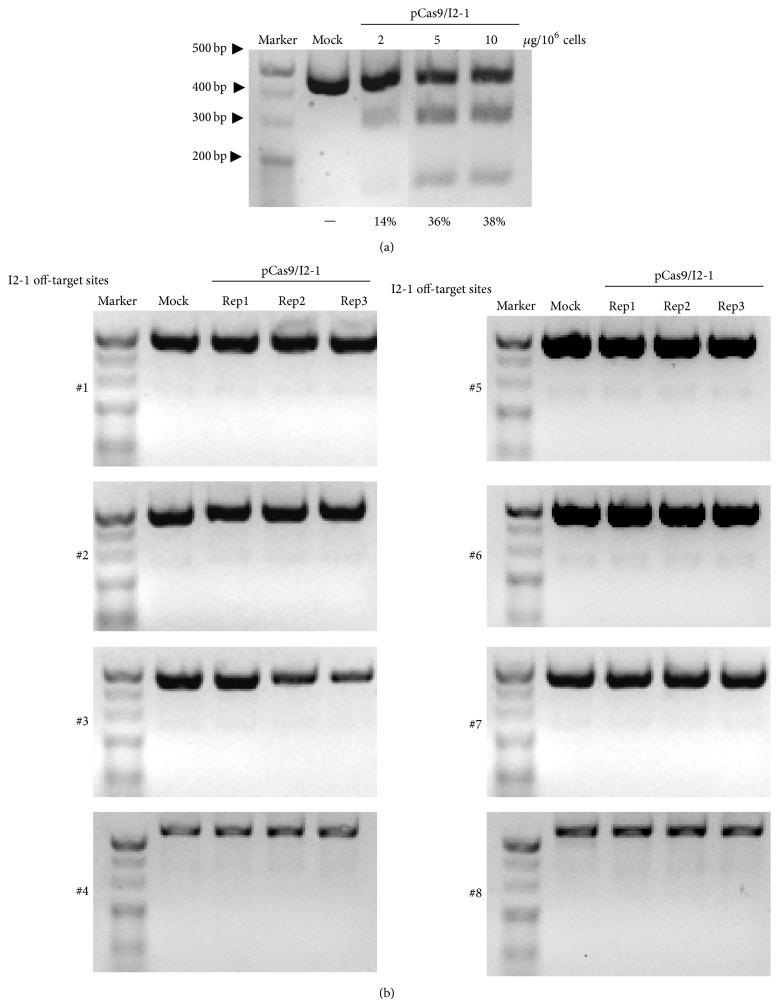
T7E1 mutation detection assays for the cleavage activity of the Cas9/I2-1 system in 293T cells. (a) On-target cleavage activities of the Cas9/I2-1 system by different concentrations. The mutated rate is quantified by ImageJ. (b) Off-target cleavage activities of the Cas9/I2-1 system (*n* = 3).

We then examined the cleavage activity of CRISPR system targeting the I2-1 site in 293T cells. Briefly, the I2-1 guide strands were annealed into the pX330 plasmid containing a Cas9/sgRNA dual expression cassette (pCas9/I2-1). 10^6^ cells in a 60 mm dish were transfected with 2, 5, and 10 *μ*g pCas9/I2-1 plasmid, respectively. Then, T7 endonuclease I (T7E1) mutation detection assay was employed to determine the mutagenesis efficiency of the Cas9/I2-1 system on the I2-1 candidate site and its 8 putative off-target sites. Briefly, DNA fragments flanking the targeted site were amplified by PCR. If CRISPR-mediated mutagenesis happened, the T7E1-cleaved fragments (2 smaller and less bright bands) could be detected besides the uncleaved full-length fragments (the larger and brighter band). The ratio of the cleaved fragments to the uncleaved fragments reflected the mutagenesis efficiency of the CRISPR system. The results showed that the Cas9/I2-1 system resulted in up to 38% mutation frequency at the targeted locus (Figure 6(a)) while no detectable cleavages were generated in the 8 putative off-target sites (Figure 6(b)).

## 4. Discussion

In this study, we describe a rational design process combining computational analyses of target uniqueness, off-target distribution, and DNA variants information to identify specific CRISPR target sites for* HBB* gene correction. We concluded that target sites in introns had 2 advantages: (i) less highly similar off-target sites at homologous genes; (ii) less off-target sites located in other exons. Indeed, exon target sites overlapping with disease-causing SNPs can be modified as mutation-specific targets [10, 11]; however, there are more than 800 reported *β*-thalassemia mutations [29], making it impractical to design and test CRISPR systems targeting each of them. The selection of intron targets without any known SNP provides a universal approach for* HBB* gene targeting. Furthermore, we suggested 2 ways to minimize possible off-target sites: (i) moderate AT content; (ii) avoid containing common regulatory elements. Based on these criteria, we recommended I2-1 (GACGAATGATTGCATCAGTGTGG) as the CRISPR target for* HBB* correction. Our T7E1 mutation detection assays confirmed that the use of this CRISPR target did not introduce significant off-target cleavages.

To facilitate precise genetic correction, the targeting specificity of the programmable artificial nuclease is crucial. Our phylogenetic analysis has shown that the human *β*-globin gene cluster is a fast evolving region by tandem duplication, thus requiring a higher recognition specificity to differentiate the cleavage activities between* HBB* and its homologous genes. The initial studies on CRISPR/Cas9 system have shown that its cleavage activity can be abolished by single-nucleotide mismatches at the sgRNA:DNA target site interface, particularly in the 3′ half [7, 8]; however, these studies were either incomplete or incomprehensive. More systematic investigations in human cells revealed that the CRISPR system could cause significant off-target effects due to the imperfectly complementary binding between sgRNA and other off-target sites with 1–3 mismatches in the genome [12–14]. Fu et al. found that the frequency of CRISPR-mediated off-target mutagenesis generally follows the known rule that target sites with more mismatches in the 3′ half proximal to the PAM are less likely to be cleaved, but not strictly [13]. There are examples that some off-target sites with up to 4 nt mismatches are cleaved at frequencies comparable to those of the intended on-target sites, as well as examples that sites with mismatches in the 3′ half are cleaved more frequently than those with mismatches in the 5′ half [13]. Thus, the targeting specificity of an sgRNA is neither easy or straightforward to be predicted.

A few bioinformatics web tools have been developed to search for specific CRISPR target sites in a given gene sequence [14, 17–19]; however, they mainly focus on analyzing the quantity of off-target sites in the genome and their distributions in the protein-coding regions. Nevertheless, the noncoding regions are not junk DNA but contain many regulatory elements essential for gene expression as well. Destruction of these elements by CRISPR-mediated off-target cleavages might also lead to severe consequences in the cells. Herein, we include analysis of the off-target distribution in transcriptional factor binding sites into our procedure. One interesting finding is that the I1-1 site containing a CCAAT sequence pattern has enriched NF-YA binding sites in its off-target set. Given that the CCAAT-binding protein NF-YA plays a critical role in hematopoietic stem cell proliferation and survival [30], the use of I1-1 in iPSCs might lead to hematopoietic defects by disrupting NF-YA regulations. Thus, we strongly suggested that the design of CRISPR targets should avoid containing any common regulatory elements, such as CCAAT box, TATA box, and GC box, which was neglected by previous studies.

## 5. Conclusion

In summary, our studies provide a standard analytical procedure to design specific CRISPR target sites between homologous genes. Here, we have showed an example how to apply this design method to identify an optimal CRISPR target site in the* HBB* gene and validate its specificity by T7E1 mutation detection assays. Geneticists who have difficulties in engineering highly homologous genes may adopt our methods to design specific CRISPR systems for their targets. This design method will also significantly enhance the safety of CRISPR-mediated genetic editing in iPSCs used for regenerative medicine and disease modeling.

## Supplementary Material

Table S1 Summary of candidate CRISPR target sites; Table S2 Summary of putative off-targets; Table S3 Primer sets used in this study.
